# Hospital Cases from the Note Book of the Late W. H. Butler, A. A. Surgeon

**Published:** 1864-04

**Authors:** 


BUFFALO

### Medical and Surgical Journal.

VOL. III. APRIL, 1864. No. 9.

ART. I.—*Hospital Cases from the Note Book of the late* W. H. BUTLER, *A. A. Surgeon.*

*Chronic Diarrhœa.*—Fernando Hobbs, aged 22 years, private Co. H, 14th N. H. Vols., entered Armory Square Hospital, March 6th, 1863. Is of sanguine nervous temperament, rather slight built, blue eyes, light hair. Says he has had diarrhœa for four months, more or less. Has now blood in the dejections, which are thin, watery and frequent, accompanied with tenesmus. He complains of pain over the small intestines, and is weak, having little appetite, though he has not lost much flesh. Treatment—acid. aromat. sulph. gutt. 20, morph. gr. $\frac{1}{4}$, every 3 hours; beef tea and milk diet.

March 8th. The acid and morph. seems to have controlled the blood in the dejections, and tenesmus, though the diarrhœa continues. ℞ Tannin and opium, gr. j, a a, every 3 hours, in pill. Treatment continued to the 12th. No change.

March 12th. ℞ Mass hydr. gr. viij.
Pulv. opii. gr. xij.
Acidum Tannicum gr. xij.
M. ft. pillulæ No. 12. Take 1 pill every 2 hours.

Complains of pain in his stomach, and vomiting food after meals. Ordered bismuth, sub nit. grs. xx, in 4 powders, to take 1 after each meal.

March 13th. The dyspeptic trouble better, but no change in the diarrhœa.

℞ Tannin, pulv. opii. a a, gr. xv.
Bismuth, sub nit. grs. xx.
M. ft. pillulæ No. 15, to take 1 pill every 3 hours.

Treatment continued until the 15th, with the addition on the 14th of enema amyli ℥iij, tannin gr. v. bed time.

March 15th. The dejections are of a greenish, stringy mucus. Turpentine stupe to bowels, and

℞ Cupri sulph. gr. vj.
Opii. pulv. gr. xij. in 13 pills; to take 1 pill every 3 hours.

March 16th. Diarrhœa seems aggravated, and the copper was stopped: the following being substituted:

℞ Ol. Olive ℥ i.
Tr. Lavend. Comp. ℥ iij.
Sach. Alba, and Acacéa a a ℥ ss.
Tinct. Opii. ℥ iij.
Aquæ ℥ iij.

To take 1 table spoonful every 3 hours.

This seemed to give the patient most relief of any medicine tried so far, the dejections being lessened nearly one-third; he appeared better on the 17th, and the remedy was continued.

March 18th. He had relapsed, and the oil and lavendar was discontinued, and chalk mixture and paregoric a a ʒij every 2 to 4 hours given, the diet being exclusively boiled milk and bread. Continued until the evening of the 21st with some improvement in the number, but not in the character of the discharges. They are thin, watery, and very offensive. This evening gave Ol. Ricini ℥ i, Tr. Opii. ʒ i, at bed time, brandy ℥ i, every 3 hours.

March 22d. Passed a comfortable night, but had several dejections ℞ Tr. opii., spts. lavend. comp. and tr. catechu a a ℥ ss. ♏, to take a tea. spoonful every 2 hours. Treatment continued on the 23d, with the addition of

Plumbi acetas gr. viij.
Morph. sulph. gr. ss.
Aquæ ℥ iv.

To be given in the morning as an enema.

Brandy ℥ i, every three hours.

March 24th. Cont. med. and brandy.

March 25. Tannin and opi, pills a a, gr. i, every 3 hours,

March 26th. Enemas of plumbi acetas, grs. iij, f. starch ℥ iij, tr. opii. ℥ i, 12 M., 6 and 8½ P. M., tannin and opii pills, 1 every 2 hours. No benefit seemed to follow the large enema of acetate of lead given on the 22d, or those of 3 grains given yesterday, combined with tr. opium, and he seems only slightly affected by large doses of opium.

March 27th. Continue tannin and opium pills every 2 hours, Ol. olive ℥ i every 4 hours; continued with benefit on the 28th, with the addition of brandy on the latter day ℥ i, every 4 hours. The dejections continuing so greenish and stringy, I had one of the medical gentlemen connected with the hospital examine a specimen under the microscope, and he reports the spots or patches noticed as floating in the aqueous discharges, as patches of the intestinal mucous surface.

March 29th. To stop all medicine except brandy, ℥ i, every 3 hours, beef tea freely.

March 30th. Nitric acid was ordered, but could not be obtained, and aromat. sulph. acid was substituted, 20 drops every 3 hours, in water. A tympanitic state of the bowels came on about mid-day, for which

Ol. Terbinth ʒ ij.

Mucil. Gum Acacia ℥ iij.

Aque Menth. ʒ iij.

To take a table spoonful every 3 hours until relieved, and to have a turpentine stupe over bowels.

March 31st. The bowels are better; little tension is apparent; continued the acid, 20 drops every 3 hours, brandy ℥ i, every 3 hours, beef tea free ly

April 1st. Has continued much the same as respects the diarrhœa for a week past. ℞ Enema amyli ℥ i, tr. opii. ʒ i, plumbi acetas gr. iij, every 3 hours; diet—tea, toast, beef tea and milk; brandy ℥ i, every 3 hours. ℞ Pulv. opii. gr. xij, plumbi acetas gr. vj, in 12 pills. To take 1 pill every 3 hours. This treatment carefully followed until the 3d. Twentieth day, ordered hydr. chlor. corrosium gr. j, que ℥ i. dose, 10 drops, 3 times daily, and to continue enemas. Continued 3 days, when some irritation seemed to be caused, and it was stopped on the 6th. The treatment to-day consisted of emplast vesical, 4x4, to bowels, enemas as above, three times, morning, noon and night.

7th. Enemas of ℥ iij. alum water, every 4 hours, (15 grs. to the ℥ i of water,) and brandy ℥ i, every 3 hours.

8th. The alum water gives him much pain, and was discontinued.

℞ S. Quinia gr. xij.
Morphia gr. iij.
Pulv. Camphor gr. x.

M. ft. chart. No. 12. One powder every 3 hours. Continued brandy. Contiuued on the 9th and part of the 10th, but the diarrhœa increasing he was ordered chalk mixture and paregoric, a a ʒ ij, every 2 hours; continued until the 14h, simply restraining an excessive diarrhœa.

℞ Mist. Creta Comp. ʒ ss.

Tinct. Catechu ʒ i.

Ol. Creosote, gutt i, (1.)

To take the above dose 3 times, daily, with Tr. Iron 10 drops, 3 times in water, ℥ ss.

15th. Says he is not so well. Treatment changed to Ol. Olive, ʒ i, every 4 hours, chalk mixture and paregoric a a ʒ ij, every 3 hours. Continued until the 20th, yesterday, the oil being diminished to 30 drops, 3 times daily. The oil being given only 3 times daily after the 15th, he had improved somewhat under this, being up and about the house. To-day only the chalk mixture and paregoric was given.

21st. Ordered oak bark ʒ i, to be steeped in water Oj, 2 hours, and to take *f* ℥ i, every 3 hours. Continued with marked benefit until the 22d, when he left for home on a furlough.

*Remarks.*—The remedies most used, and which seemed to be of most benefit, were sweet oil, the combination of chalk mixture and paregoric, and the oak bark tea.

